# A Medical Swindler

**Published:** 1877-10-20

**Authors:** 


A MEDICAL SWINDLER.

Chas. H. Lothrop, M. D., of Lyons, Iowa, kindly informs us of the doings of one A. G. Walter, alias Willard, alias H. Bayrenz, etc., etc. Said Walter has been going round claiming to be an agent of the "U. S. College of Surgeons, Washington, D. C.," an institution endowed by the U. S. Government for the special purpose of treating Paralytics. Parties afflicted with this disease by paying a small sum could receive treatment at this humane institution. The testimonials as to this institution, which really has no existence, furnished by the shrewd swindler, were such as would deceive any one. He thus filched thirty-one dollars from Dr. L., who, however, had his suspicions aroused, and took steps which resulted in the arrest of the scoundrel, who *is now* safely lodged in De-Witt jail.

